# Entanglement of Signal Paths via Noisy Superconducting Quantum Devices

**DOI:** 10.3390/e25010153

**Published:** 2023-01-12

**Authors:** Wenbo Shi, Robert Malaney

**Affiliations:** School of Electrical Engineering & Telecommunications, University of New South Wales, Sydney, NSW 2052, Australia

**Keywords:** quantum routing, quantum entanglement, quantum communications, quantum error correction

## Abstract

Quantum routers will provide for important functionality in emerging quantum networks, and the deployment of quantum routing in real networks will initially be realized on low-complexity (few-qubit) noisy quantum devices. A true working quantum router will represent a new application for quantum entanglement—the coherent superposition of multiple communication paths traversed by the same quantum signal. Most end-user benefits of this application are yet to be discovered, but a few important use-cases are now known. In this work, we investigate the deployment of quantum routing on low-complexity superconducting quantum devices. In such devices, we verify the quantum nature of the routing process as well as the preservation of the routed quantum signal. We also implement quantum random access memory, a key application of quantum routing, on these same devices. Our experiments then embed a five-qubit quantum error-correcting code within the router, outlining the pathway for error-corrected quantum routing. We detail the importance of the qubit-coupling map for a superconducting quantum device that hopes to act as a quantum router, and experimentally verify that optimizing the number of controlled-X gates decreases hardware errors that impact routing performance. Our results indicate that near-term realization of quantum routing using noisy superconducting quantum devices within real-world quantum networks is possible.

## 1. Introduction

Today’s quantum computing devices are regarded as Noisy Intermediate-Scale Quantum (NISQ) devices due to their intrinsic errors and limited inter-qubit connections—issues which present a challenge for implementing quantum computation tasks [1]. Sources of error include quantum gate errors, measurement errors, decoherence errors, and cross-talk errors. Quantum gate errors include single-qubit and 2-qubit gate errors, accumulating over a long sequence of quantum gates. Measurement errors incorrectly determine a qubit in the |0〉 state as the |1〉 state, and vice versa. Decoherence errors are caused by the short thermal relaxation and dephasing times. Cross-talk errors occur when multiple quantum operations are executed in parallel and corrupt the quantum states of qubits. Henceforth, we term all the above-mentioned errors as ‘intrinsic’ errors. To identify, mitigate, and even correct these errors on quantum signals and gates, many quantum error correction techniques have been pursued, chief among them being Quantum Error-Correcting Codes (QECCs) [2,3,4,5,6,7,8,9,10,11]. The implementation of QECCs on NISQ devices is still in its infancy—but some small performance improvements have been reported [12].

In this work, we focus on the NISQ devices provided by the IBM Quantum Experience (IBM Q), a cloud platform available to the wider community [13]. The IBM Q provides access to multiple superconducting quantum devices, ranging from a 5-qubit device through to a 127-qubit device [13]. Our specific task here will be to investigate the performance of *quantum routing* on the NISQ devices provided by the IBM Q with a focus on low-complexity devices, referring to those devices with seven qubits or less. Due to their lower complexity, these devices are likely to be the first to be deployed in real-world quantum networks.

Quantum routers are considered an essential element of a future quantum network [14]. Indeed, one of the most important distinguishing characteristics of a quantum network is its ability to route quantum signals over multiple paths simultaneously [15,16,17,18]. This multi-path quantum routing should not be confused with multi-directional broadcast in classical networks (not possible for quantum signals due to the no-cloning theorem), but rather a quantum-only phenomenon in which the path taken by the quantum signal is a *coherent superposition* of multiple paths [16]. This superposition of paths is unique to quantum communication and it represents a startling application of quantum entanglement. Although all benefits of this application for communications have yet to be crystallized, some firm uses are already known. For example, in communications enabled via quantum routing, interference occurring on different paths can result in throughput enhancement [19,20,21], as can the use of alternative path orderings (a slight variation of quantum routing) [22,23,24]. Quantum Random Access Memory (QRAM) [25,26,27] via quantum routing is another application important to the communication space. Beyond communications, quantum routing can also assist in the realization of quantum machine learning [28,29]. More specifically, the technique of quantum routers provides the foundation of exponential speedup in large data processing [25]. Beyond applications in communications, the creation of QRAM, and assistance to quantum machine learning, we anticipate a wider range of use-cases will be discovered as quantum routing starts to be deployed in real networks.

However, as discussed above, the quantum signal is transmitted through a noisy quantum device, and various errors will be introduced via the environment. We are focused on determining the performance of quantum routing under such errors, as well as in an application of quantum routing, namely QRAM. We will also investigate the use of a QECC code within the context of quantum routing. Our previous work [30] experimentally implemented a quantum router with an error correction technique, taken from [31], which offered a solution specifically designed for reversing a weak measurement on a qubit. The results of [30] showed, however, that the error correction technique, in the context of quantum routing, was only useful for very specific quantum channels—rendering it of no value for generic (or unknown) channel conditions. In this current work, we utilize a more sophisticated error correction technique. Specifically, we embed a robust QECC within the quantum router that can correct for any single qubit error, independent of the quantum noise model. As we shall see, although such error correction is not viable on current low-complexity NISQ devices provided by the IBM Q, our work highlights the pathway forward to more robust error-corrected quantum routing.

Our novel contributions in this work are as follows: (i) We first benchmark quantum routing performance using a currently available 7-qubit NISQ device, assuming noiseless quantum signals. (ii) Using such quantum routing we then implement, for the first time, a viable QRAM. (iii) We then evaluate the performance of a quantum router embedded within a 5-qubit QECC suitable for any single-qubit error channel. In doing this, we consider the intrinsic errors as well as errors that mimic the effects of a noisy quantum channel. (iv) With regard to the quantum error-corrected routing performance of NISQ devices, we experimentally demonstrate the significance of minimizing the number of control gates utilized, and identify the importance of the qubit-coupling map of superconducting quantum devices for realizing a quantum routing process over noisy channels.

The rest of this paper is organized as follows. In Section 2, we introduce the basic principles of the quantum router, as well the required quantum circuits for the adopted QECC. Section 3 reports the experimental results of the quantum router with and without QECC. Insights for improving the performance of the NISQ devices are provided in Section 3, and Section 4 concludes the work.

## 2. Principles

### 2.1. Quantum Routing

**Figure 1:**
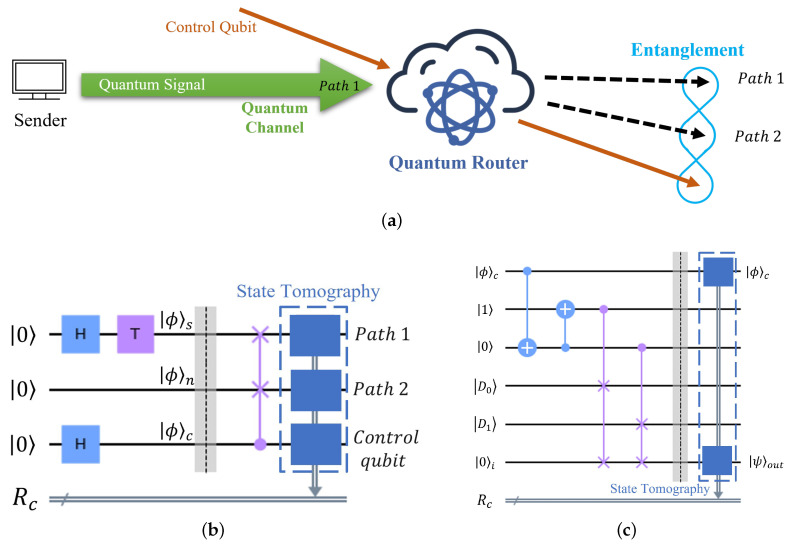
(**a**) Schematic diagram illustrating the principle of a quantum router. A sender prepares and sends a quantum signal to the quantum router via a quantum channel, and a control qubit directs the quantum signal’s path based on the control information it stores. The output of the quantum router is an entanglement between the control qubit and the two paths. (**b**) Quantum circuit of a quantum router with state tomography. H stands for the Hadamard gate, and T is the phase gate that introduces a π/4 phase. All qubits start from the |0〉 state, and the second qubit initially is ${|\phi \rangle }_{n}={|0\rangle }_{n}$. The 3-qubit gate in purple is the controlled-swap gate, which exchanges the two quantum states (represented by the two crosses) when the control qubit (represented by the solid circle) is in the |1〉 state. The state tomography reconstructs the density matrix of the quantum router’s output. $R_{c}$ represents a classical register that contains the quantum measurement results of the state tomography. (**c**) Quantum circuit of a QRAM with state tomography. The 2-qubit gate is the CX gate (Controlled-X), which is applied to a control (represented by the solid circle) and a target qubit (represented by the circle with a plus sign).

In the following, the identity matrix is denoted *I*, and the Pauli matrices are $X=\left[\begin{array}{cc} 0 & 1\\ 1 & 0 \end{array}\right]$, $Y=\left[\begin{array}{cc} 0 & -i\\ i & 0 \end{array}\right]$, and $Z=\left[\begin{array}{cc} 1 & 0\\ 0 & -1 \end{array}\right]$. I, X, Y, and Z (non-italic) denote their corresponding gates. A schematic diagram of the principle of a quantum router is shown in Figure 1a, and a quantum circuit of the quantum router with state tomography is shown in Figure 1b—henceforth, we refer to this circuit as the ‘router circuit.’ Three qubits start from the |0〉 state in the router circuit, and then they are prepared to a signal qubit ${|\phi \rangle }_{s}$, a ‘blank’ qubit ${|\phi \rangle }_{n}$, and a control qubit ${|\phi \rangle }_{c}$. The state ${|\phi \rangle }_{s}$ carries the quantum signal and is injected into the quantum router via path 1. This quantum signal is written as

(1) $${|\phi \rangle }_{s}=\alpha_{s}{|0\rangle }_{s}+\beta_{s}{|1\rangle }_{s}={\cos\left(\pi /4\right)|0\rangle }_{s}+e^{i\pi /4}\sin\left(\pi /4\right){|1\rangle }_{s},$$

where $\alpha_{s}$ and $\beta_{s}$ are complex numbers satisfying $|\alpha_{s}{|}^{2}+{\left|\beta_{s}\right|}^{2}=1$. We define $\alpha_{s}=\cos\left(\pi /4\right)$ and $\beta_{s}=e^{i\pi /4}\sin\left(\pi /4\right)$, and note that ${|\phi \rangle }_{s}$ can be prepared by sequentially operating the Hadamard gate and the T gate (π/4 phase gate) on the |0〉 state. The state ${|\phi \rangle }_{n}={|0\rangle }_{n}$, which is initially at path 2, represents a qubit that carries no signal information. The state ${|\phi \rangle }_{c}$ contains the control information which directs the path of ${|\phi \rangle }_{s}$ and is prepared by the Hadamard gate from the |0〉 state. The state ${|\phi \rangle }_{c}$ can be expressed as

(2) $$\begin{array}{cc} {|\phi \rangle }_{c} & =\alpha_{c}{|0\rangle }_{c}+\beta_{c}{|1\rangle }_{c}={(|0\rangle }_{c}+{|1\rangle }_{c})/\sqrt{2}, \end{array}$$

where $\alpha_{c}$ and $\beta_{c}$ are again complex numbers satisfying a normalization constraint. The input of the quantum router can then be written as $|\Phi \rangle ={|\phi \rangle }_{s}{|\phi \rangle }_{n}{|\phi \rangle }_{c}$, and the output of the quantum router is written as

(3) $$\begin{array}{cc} {|\Phi \rangle }_{f} & =\alpha_{c}{|\phi \rangle }_{s}{|\phi \rangle }_{n}{|0\rangle }_{c}+\beta_{c}{|\phi \rangle }_{n}{|\phi \rangle }_{s}{|1\rangle }_{c}, \end{array}$$

which displays an entanglement between the control qubit and the two output paths. The signal qubit, ${|\phi \rangle }_{s}$, is in the path 1 when ${|\phi \rangle }_{c}={|0\rangle }_{c}$ and is routed to the path 2 when ${|\phi \rangle }_{c}={|1\rangle }_{c}$.

**Figure 2:**
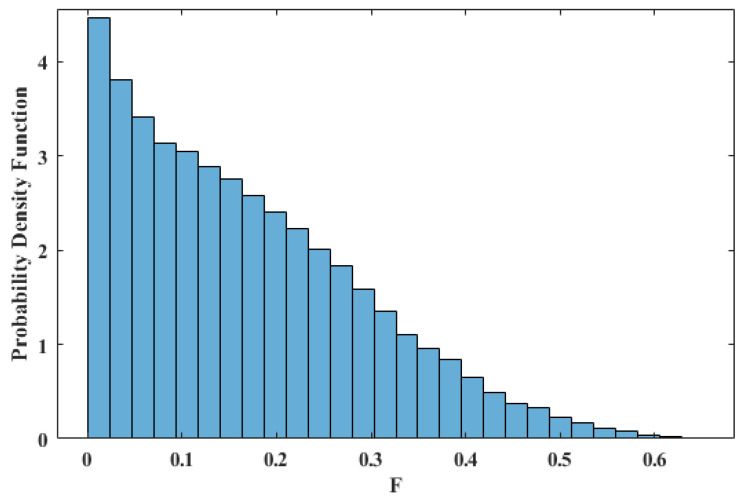
Probability density function of *F* between ρ and $\rho_{r}$ obtained from 100,000 samples.

Let us define $\rho ={|\Phi \rangle }_{f}\langle \Phi |$, which represents the final state after passing through a perfect (zero error) router—henceforth, we refer to this as the ‘theoretical’ density matrix. $\rho_{1}$ is the density matrix of ${|\Phi \rangle }_{f}$ after passage through the real router, and we refer to this as the ‘experimental’ density matrix. It is reconstructed by state tomography applied on the three qubits, as demonstrated in Figure 1b. The detailed tomography procedure is specified in Appendix A. We choose the entanglement fidelity *F* between ρ and $\rho_{1}$ as our performance metric, which is calculated as

(4) $$\begin{array}{cc} F & ={\left(\mathrm{Tr}\sqrt{\sqrt{\rho }\;\;\rho_{1}\sqrt{\rho }}\right)}^{2}, \end{array}$$

where Tr represents the trace operation. When we run our experiments, it will be useful to have a ‘comparison’ fidelity with which to compare our results. Figure 2 shows a distribution of the fidelity between ρ and $\rho_{r}$, determined from Equation (4), where $\rho_{r}$ is a 3-qubit system uniformly sampled from the space of all possible 3-qubit systems. (Here, we used the fact that a generic 3-qubit state can be expressed with only 5 terms with the help of the canonical 5-term decomposition, that is, $|\Delta \rangle =\lambda_{0}|000\rangle +\lambda_{1}e^{i\varphi }|100\rangle +\lambda_{2}|101\rangle +\lambda_{3}|110\rangle +\lambda_{4}|111\rangle$, where $\lambda_{i},\varphi \in R$, $\sum \lambda_{i}^{2}=1$, and φ∈(0,π) is a phase term [32]). From this, we see that the probability of randomly selecting a state with F>0.5 is less than 1.25%. As an additional comparison, we note the averaged fidelity between ρ and a 3-qubit product state uniformly sampled from {|000〉, |001〉, ⋯, |111〉}, is F=0.125.

The quantum router directs the signal qubit along the two paths in a coherent superposition, a process that can be utilized for QRAM. As opposed to classical RAM, QRAM can access the information stored in memory in superposition. There are mainly two QRAM structures: the conventional fanout structure [33] and the ‘bucket brigade’ structure [25]. In the fanout QRAM, each ‘address’ qubit changes the states of all ‘routing nodes’ that constitute a binary tree architecture. (The binary tree architecture is a widely used data structure for RAM and it maintains binary relationships among memory elements). The routing nodes are qubits used for the decision-making in the binary tree architecture to access memory elements (see [26]). The fanout QRAM is vulnerable to decoherence errors due to its working principle, limiting its scalability. In contrast, the bucket brigade QRAM has a higher noise resistance than the fanout QRAM since the address qubits only change the states of the routing nodes needed for accessing memory elements [27,34].

Based on the circuit for the bucket brigade QRAM in [26], we design a quantum circuit of our QRAM (the ‘QRAM circuit’) with one address qubit and two memory elements, as shown in Figure 1c. In our QRAM, what was termed previously as the control qubit and is now referred to as the address qubit, ${|\phi \rangle }_{c}$, which is a superposition of two addresses (0 and 1). The two qubits initialized to the |1〉 and |0〉 states are the routing nodes (referred to above) that help to access the memory elements, $|D_{0}\rangle$ and $|D_{1}\rangle$, storing bits or qubits. We use the last qubit initialized as ${|0\rangle }_{i}$ in the QRAM circuit to store the accessed memory elements. We define that the input and the output of our QRAM is ${|\Psi \rangle }_{i}$ and ${|\Psi \rangle }_{f}$, respectively, which can be expressed as

(5) $$\begin{array}{cc} {|\Psi \rangle }_{i} & ={|\phi \rangle }_{c}{|0\rangle }_{i}\overset{\mathrm{QRAM}}{\to }{|\Psi \rangle }_{f}={|\phi \rangle }_{c}{|\psi \rangle }_{out}=\alpha_{c}{|0\rangle }_{c}|D_{0}\rangle_{out}+\beta_{c}{|1\rangle }_{c}{|D_{1}\rangle }_{out}. \end{array}$$

### 2.2. Five-Qubit QECC

**Figure 3:**
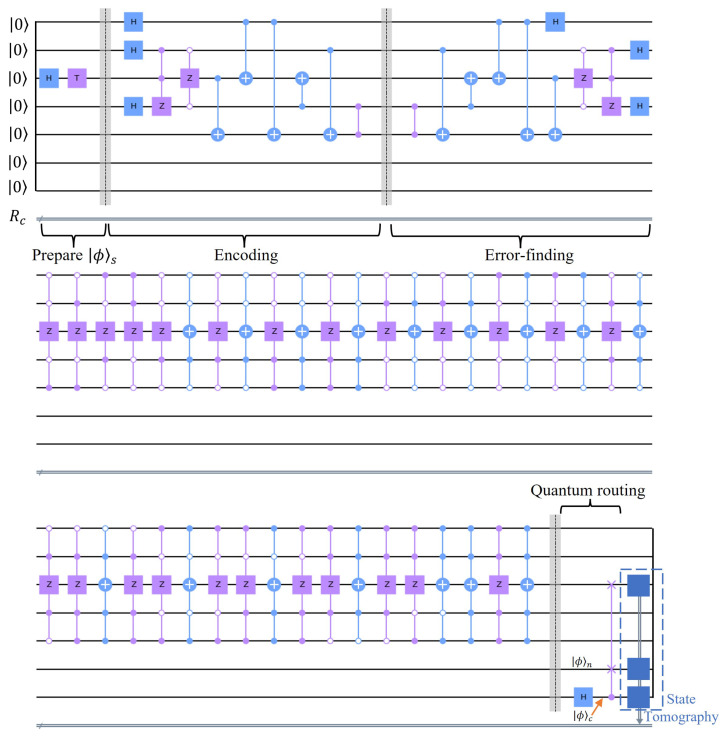
Quantum circuit of the 5-qubit QECC embedded within the quantum router with state tomography. The first part of this quantum circuit is the state preparation of ${|\phi \rangle }_{s}$ on the third qubit (counting from the top), followed by the encoding, error-finding, error correction, and quantum routing. The first five qubits, except for the third qubit, are ancillary qubits for encoding, the sixth qubit is ${|\phi \rangle }_{n}$, and the last qubit is prepared as ${|\phi \rangle }_{c}$. Z stands for the Pauli-Z gate, the 2-qubit gate with two solid circles is the CZ (controlled-Z) gate. The 3-qubit and the 5-qubit gates are either a multi-CX or a multi-CZ gate, whose solid and hollow circles indicate that the control state is the |1〉 or |0〉 state, respectively. Note that the row being continued across the three blocks is the continuation of the previous row.

The QECC code we adopt is that of [35], which encodes the quantum signal by 5 qubits, requires no post-selections or stabilizer measurements. The best-known 5-qubit QECC is the [[5,1,3]] code [36], which requires not only 5 qubits for encoding but also four ancillary qubits for stabilizer measurements (requires nine qubits in total). The 5-qubit QECC [35] utilized for this research only requires 5 qubits in total, and it permits corrections of generic single-qubit errors. A quantum circuit of the QECC (the ‘QECC circuit’) embedded within the quantum router is demonstrated in Figure 3. The first five qubits (counting from the top) in the QECC circuit are utilized for an encoding logic given by

(6) $$\begin{array}{cc} |0\rangle & \to \frac{1}{\sqrt{8}}\left(|00000\rangle -|01111\rangle -|10011\rangle +|11100\rangle +|00110\rangle +|01001\rangle +|10101\rangle +|11010\rangle \right),\\ |1\rangle & \to \frac{1}{\sqrt{8}}\left(|11111\rangle -|10000\rangle +|01100\rangle -|00011\rangle +|11001\rangle +|10110\rangle -|01010\rangle -|00101\rangle \right). \end{array}$$

The encoded qubit is the third qubit, which is prepared as ${|\phi \rangle }_{s}$ before the encoding. After the encoding, the five qubits are sent to the quantum router via a noisy quantum channel, which introduces a generic single-qubit error. We utilize a unitary transform *U* to represent this single-qubit error given by

(7) $$U=\left[\begin{array}{cc} c_{0} & c_{1}\\ c_{2} & c_{3} \end{array}\right]=\frac{c_{0}+c_{3}}{2}I+\frac{c_{1}+c_{2}}{2}X+\frac{c_{2}-c_{1}}{2i}Y+\frac{c_{0}-c_{3}}{2}Z,$$

where $c_{0}$, $c_{1}$, $c_{2}$, and $c_{3}$ are complex numbers and satisfy the requirements of a unitary matrix.

Once the quantum router receives the qubits, it realizes the error-finding, error-correction, and quantum-routing processes. After the error-finding process, quantum measurements should be applied to the first, second, fourth, and fifth qubits to find syndromes that indicate the exact error that occurred on any of the five qubits, as summarized in Table 1. However, due to the limitations of current 7-qubit superconducting quantum devices, we cannot apply quantum gates based on the quantum measurement results. Instead, we implement multi-controlled gates conditioned on the states of those four qubits, as shown in Figure 3. Note that the multi-controlled gates realizing the error correction process are equivalent to applying the X and Z gates on the third qubit conditioned on the measurement results of the four qubits [37].

The sixth qubit is ${|\phi \rangle }_{n}$, and the last qubit is prepared as ${|\phi \rangle }_{c}$ by the Hadamard gate. Upon completing the quantum routing process, we implement the state tomography on the third and the last two qubits to reconstruct $\rho_{1}$, thereby allowing us to determine *F* (see Appendix A).

## 3. Experimental Results

### 3.1. Quantum Routing without QECC

Our experiments are implemented on the seven-qubit *ibmq_jakarta*, and all the above quantum circuits are designed using IBM’s open-source software development kit—the Quantum Information Science toolKit (Qiskit) [38]. As specified in Appendix B, executing a circuit on a quantum device requires a process named *Transpilation*, and we refer to the circuit after the transpilation as a ‘transpiled’ circuit. The general transpilation process includes steps which are stochastic in nature, e.g., the number of gates resulting from the transpilation (see Appendix B). However, it is possible to generate reproducible and deterministic transpiled circuits—and we adopt this approach here (ensuring the minimum number of CX gates are used). In addition, all measurement results returned from the quantum device are first processed (using a Qiskit package) so as to mitigate measurement errors, one type of intrinsic error. The logic of measurement error mitigation is that the package builds a calibration matrix based on the measurement outcomes of basis states (the |0〉 and |1〉 states) for all measured qubits. Then, the inverse of this calibration matrix is applied to the measurement results obtained from the transpiled circuits, eliminating measurement errors in the ideal case.

**Figure 4:**
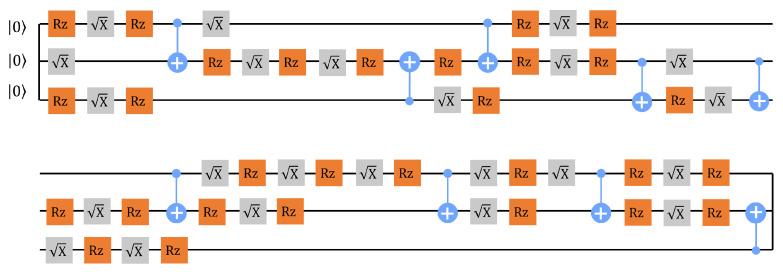
A transpiled circuit of the router circuit. Note that the state tomography is not included here, and 9 CX gates are involved. The quantum gates included in this transpiled circuit are basis gates that can be physically operated on the *ibmq_jakarta*.

To benchmark the performance of the quantum router on the *ibmq_jakarta*, we implement two quantum routing experiments that verify the quantum nature of the quantum router and the preservation of ${|\phi \rangle }_{s}$, respectively. We refer to these experiments as ‘*Experiment-1*’ and ‘*Experiment-2*,’ respectively, and we assume that the quantum channel introduces zero noise in these two experiments, which means that only the intrinsic errors of the *ibmq_jakarta* are included. In *Experiment-1*, we execute the router circuit to confirm the quantum nature by experimentally demonstrating the generation of ${|\Phi \rangle }_{f}$. The transpiled circuit of the router circuit is demonstrated in Figure 4, and it includes 9 CX gates. There are 27 projective measurements in the state tomography (see Appendix A), which is applied at the end of this transpiled circuit. We refer to one single execution of a circuit as one ‘shot’. That is, when a circuit executes 100 shots, say, the circuit is executed 100 times. The transpiled circuit executes 100,000 shots for each projective measurement, which is the maximum number of shots for the *ibmq_jakarta*. The results of the projective measurements returned from the *ibmq_jakarta* are first processed by the measurement error mitigation and then utilized for calculating *F*. The entire procedure from the transpilation to the calculation of *F* is referred to as a ‘run.’ Several runs with the same transpiled circuit are taken, from which the averaged *F* is adopted as our performance metric.

**Figure 5:**
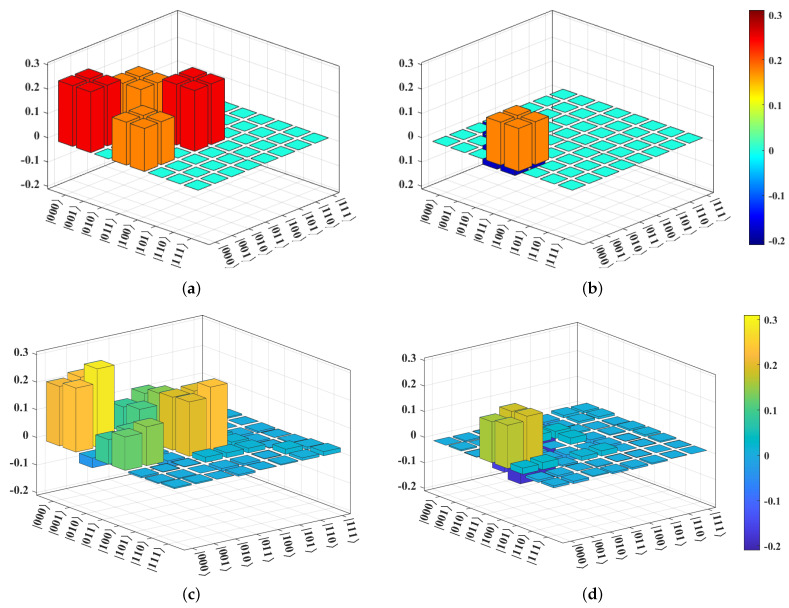
Theoretical and experimental density matrices of ${|\Phi \rangle }_{f}$. (**a**,**b**) represent the real and imaginary parts of the theoretical density matrix, ρ, respectively. (**c**,**d**) show the real and imaginary parts of the experimental density matrix, $\rho_{1}$, respectively. The entanglement fidelity, *F*, between ρ and $\rho_{1}$ is 0.85.

**Figure 6:**
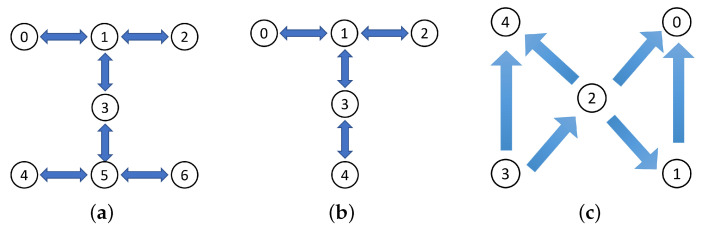
Coupling maps of the *ibmq_jakarta* (**a**), the *ibmq_quito* and *ibmq_belem* (**b**), and the *ibmqx4* (**c**). The two-way arrows in (**a**,**b**) represent that the CX gate can be implemented between the two pointed qubits in both directions. The one-way arrows in (**c**) indicate that the CX gate can only be implemented in one direction (the arrowheads point to the target qubits). In the connected qubit pair labeled by 0 and 1 in (**c**), for example, qubit 1 can only act as the control qubit of the CX gate with the qubit 0 as the target qubit.

The comparison of ρ and $\rho_{1}$ is demonstrated in Figure 5. Here, after ten runs, an average F=0.85 is determined. We conclude that the two output paths of the quantum router are entangled, and the quantum nature is verified on the quantum device. We emphasize that four specific experimental setups help improve the performance of the *ibmq_jakarta*. These are the following: (i) We chose three physically connected qubits (qubits labeled by 0, 1, and 3 in Figure 6a) of the quantum device with the lowest single-qubit and two-qubit error rates to implement *Experiment-1*. (The *ibmq_jakarta* is calibrated daily, and the error rates may change over time). (ii) We utilized the transpiled circuit with the lowest number of CX gates. (iii) We executed the transpiled circuit with the maximum shots. (iv) The measurement error mitigation was applied to the data returned from the quantum device.

As the errors in two-qubit gates and single-qubit gates are of the order $10^{-2}$ and $10^{-4}$, respectively, optimizing the number of CX gates is a helpful way to reduce quantum gate errors [39]. We implement the quantum router with another transpiled circuit which includes 14 CX gates instead of 9 CX gates, and with other experimental setups unchanged, we find that *F* decreases to 0.68. This phenomenon emphasizes the significance of lowering the number of CX gates with regard to routing performance. We also note that *F* drops from 0.85 to 0.79 if the measurement error mitigation is removed, highlighting the importance of this step.

**Figure 7:**
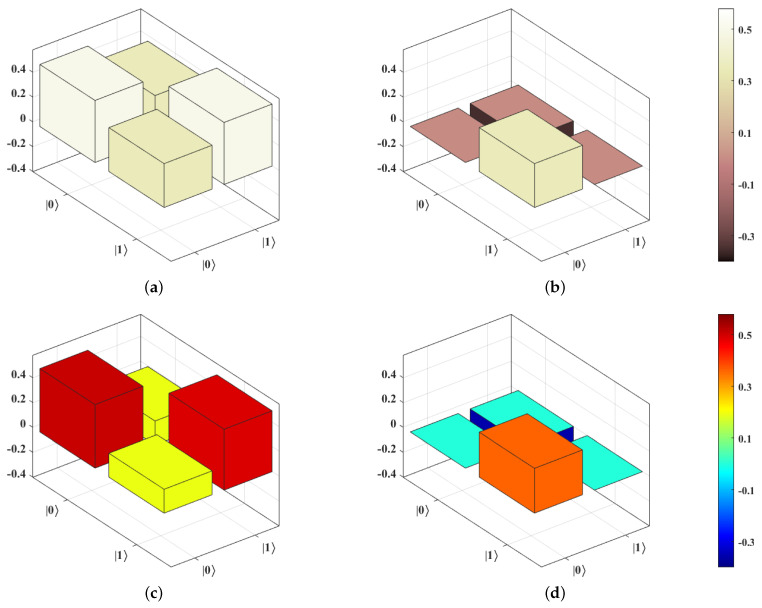
Theoretical and experimental density matrices of ${|\phi \rangle }_{s}$ after the quantum routing process with ${|\phi \rangle }_{c^{\prime }}={|1\rangle }_{c}$. (**a**,**b**) represent the real and imaginary parts of the theoretical density matrix, σ, respectively. (**c**,**d**) show the real and imaginary parts of the experimental density matrix, $\sigma_{1}$, respectively. The state fidelity, $F_{S}$, between σ and $\sigma_{1}$ is 0.89.

We now review the preservation of the quantum signal. *Experiment-2* utilizes the router circuit but with a slight modification: the control qubit is set as ${|\phi \rangle }_{c^{\prime }}={|1\rangle }_{c}$ by applying an X gate instead of the Hadamard gate on the |0〉 state. Moreover, a single-qubit state tomography is applied to the qubit in path 2 to check whether its state is ${|\phi \rangle }_{s}$. We use the state fidelity $F_{S}={\left(Tr\sqrt{\sqrt{\sigma }\;\;\sigma_{1}\sqrt{\sigma }}\right)}^{2}$ between $\sigma ={|\phi \rangle }_{s}\langle \phi |$ and $\sigma_{1}$ as our performance metric, where now we refer to σ as the theoretical density matrix (no errors) of ${|\phi \rangle }_{s}$ and $\sigma_{1}$ as the experimental density matrix of ${|\phi \rangle }_{s}$ reconstructed by the single-qubit state tomography. Similar to our previous discussion, the modified router circuit is transformed into a transpiled circuit with 9 CX gates. $F_{S}$ is averaged from ten runs and is found to be 0.89, as demonstrated in Figure 7. This result implies that the quantum signal is well preserved after the quantum routing process on the *ibmq_jakarta*.

We return to the full implementation of the quantum router (*Experiment-1*) on the *ibmq_jakarta*, whose coupling map is illustrated in Figure 6a, and where we obtained an averaged result of F=0.85. However, Behera et al. [18] demonstrated a quantum router on the *ibmqx4* (its coupling map is shown in Figure 6c) and obtained an *F* of approximately 0.98. We name their router the ‘BRGP’ router to differ from our router shown in Figure 1b, as these two routers utilize different quantum states (The BRGP router sets the control and signal qubits in states ${(|0\rangle }_{c}-e^{i\pi /4}{|1\rangle }_{c})/\sqrt{2}$ and ${\cos\left(\pi /8\right)|0\rangle }_{s}+\sin\left(\pi /8\right){|1\rangle }_{s}$, respectively. The control qubit is prepared by sequentially applying H, S, T, and S gates on the |0〉 state, where the S gate is a phase gate that introduces a π/2 phase. Similarly, the signal qubit is prepared by sequentially implementing H, T, H, and S gates on the |0〉 state. The BRGP router defines that the blank qubit is in the ${(|0\rangle }_{n}+{|1\rangle }_{n})/\sqrt{2}$ state. In contrast, in the router of Figure 1b, we utilize ${|0\rangle }_{n}$ to represent the blank qubit, meaning that it is an ancillary qubit without any information in the quantum router). and devices. As the *ibmqx4* is retired, we reproduce the BRGP router on the *ibmq_quito* and *ibmq_belem*, which are five-qubit quantum devices sharing a coupling map (as shown in Figure 6b) close to the *ibmqx4*’s map. The experimental results we obtained from the *ibmq_quito* and *ibmq_belem* were F=0.85 and 0.79, respectively. Thus, we conclude that F=0.85 obtained in *Experiment-1* is close to the best outcome for a quantum router built using currently available small-qubit superconducting quantum devices.

The disparity of *F* between 0.85 and 0.98 likely demonstrates the importance of the coupling map for the quantum device acting as the quantum router. The coupling map of the *ibmqx4* ensures that every qubit is connected to two or four qubits, and we can find that qubits 0, 1, and 2 (same for qubits 2, 3, and 4) are connected to each other. The controlled-swap gate is a three-qubit quantum gate that realizes the quantum routing process, which therefore benefits from this topology. We also note that in this topology, the CX gate can only be implemented in one way on the *ibmqx4* (see Figure 6). However, this disadvantage is somewhat compensated by the fact that on the *ibmqx4* topology, an inverse-CX gate can be implemented (by adding four Hadamard gates [40]). Even though *ibmq_quito* and *ibmq_belem* can implement the CX gate in both directions, they do not have a similar three-qubit topology, which leads to the implementation of the controlled-swap gate requiring multiple swap gates during the transpilation. The introduction of swap gates causes extra quantum gate errors, thus decreasing the performance of the quantum router. In summary, having a coupling map with at least three qubits physically connected is significant for a superconducting quantum device that hopes to act as the quantum router.

Additionally, we implement another experiment (‘*Experiment-3*’) that executes the QRAM circuit. We first consider the case where the memory stores quantum information, which means that $|D_{0}\rangle$ and $|D_{1}\rangle$ are set as two arbitrary qubits. We utilize the fidelity $F^{\prime }={\left(Tr\sqrt{\sqrt{\eta }\;\;\eta_{1}\sqrt{\eta }}\right)}^{2}$ as our performance metric, where now we refer to $\eta ={|\Psi \rangle }_{f}\langle \Psi |$ as the theoretical density matrix (no errors) of ${|\Psi \rangle }_{f}$ and $\eta_{1}$ as the experimental density matrix of ${|\Psi \rangle }_{f}$ reconstructed by state tomography. The experimental result is $F^{\prime }=0.86$, which is an averaged result from ten runs. This result indicates that QRAM with quantum memory is feasible on current small-qubit NISQ devices. We next consider the case where the memory stores classical information. In this case, the experimental result is slightly higher ($F^{\prime }=0.88$) than the quantum case since fewer basis gates are required for initializing the memory elements $|D_{0}\rangle =|1\rangle$ and $|D_{1}\rangle =|0\rangle$. Entanglement distillation protocols are designed so that higher entanglement can be created from an ensemble of states possessing lower entanglement [41,42]. The experimental results for our QRAM, $F^{\prime }$, are all above 0.5, which is high enough to implement a viable distillation process. Therefore, we conclude that our QRAM is viable. To the best of our knowledge, no other experimental realizations of QRAM using quantum routing have been achieved.

### 3.2. Quantum Router with QECC

We now implement an experiment that executes the QECC circuit—we refer to this experiment as ‘*Experiment-4*’. Although we will find, not surprisingly, that full-blown QECC for quantum routing is not feasible on *current* small-qubit superconducting NISQ devices, our results highlight the pathway towards error-corrected versions of quantum routing.

The transpiled circuit of the QECC circuit has thousands of CX gates and is too complex to illustrate here. Due to the long run time in the quantum device for the QECC circuit, we take *F* averaged from only four runs as our performance metric. While *Experiment-1* implemented the quantum router on the quantum device with intrinsic errors only, *Experiment-4* considers the QECC applied to generic single-qubit errors. We consider a noise model in which a noisy quantum channel introduces one generic single-qubit error that can be represented by *U*, as explained in Equation (7). In this noise model, U={G,X,Y,Z}, where *G* is a 2×2 unitary matrix randomly generated (different in each of the runs). In *Experiment-4*, *U* is manually applied to ${|\phi \rangle }_{s}$ when the QECC is not considered. When the QECC is considered, *U* is randomly applied to any of the five qubits by codes after the encoding.

**Figure 8:**
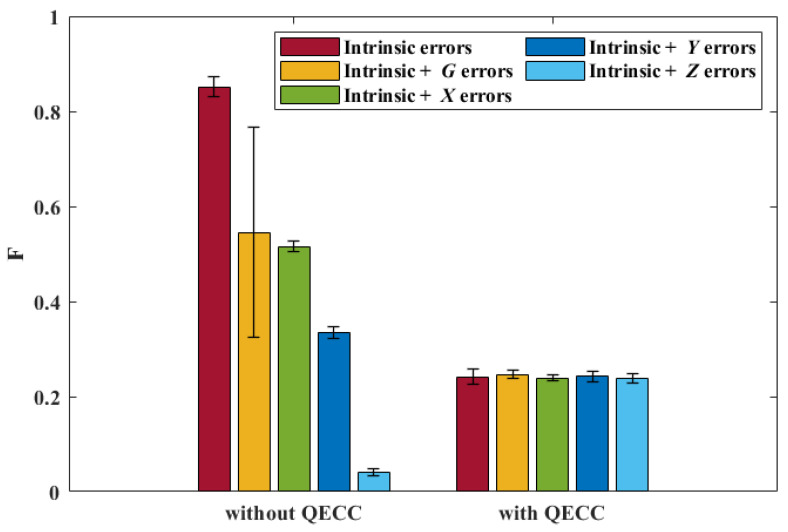
*F* of the quantum router without and with the QECC.

The results of *Experiment-4* are demonstrated in Figure 8, where each bar shows the averaged *F* from four runs (the error bars represent the standard deviation). As we can see, only when U=Z do we find that *F* is improved after considering the QECC. Indeed, we see that *F* is effectively constant with the QECC included, no matter which type of errors are included. Focusing on the intrinsic-errors-only bars, this demonstrates that *F* decreases substantially after introducing the QECC even when only intrinsic errors are present. The reason for this phenomenon is that thousands of basis gates are introduced after the transpilation due to the QECC, and thus, the intrinsic errors (mainly quantum gate errors) accumulate. The transpilation decomposes all of the five-qubit gates in Figure 3 (those gates taking five qubits as an input) into thousands of basis gates, while the remaining quantum gates only transpile to hundreds of basis gates. We conclude that the adopted QECC is not effective when working on current small-qubit superconducting NISQ devices. This is, in part, evidenced by noticing that after introducing the QECC *F* is only 0.24. The probability of the ‘comparison’ fidelity, discussed earlier, being larger than 0.24 is 27%, as shown in Figure 2.

Let us summarize our findings regarding QECC. We have shown how quantum routing can be performed, with useful fidelity, on currently available small-qubit NISQ devices—under the assumption that the quantum signals traversing the network arrive at the router in a noiseless condition. However, we have also shown how correction of arriving noisy quantum signals within the router is plagued by the very large number of CX gates that arise from the transpilation process required to run the QECC circuits alongside the routing circuits. We have provided evidence that a reduction in the number of these gates is the pathway to full-blown QECC quantum routing. In this regard, we note the correction techniques based on self-mitigation via Trotter circuits that several groups have proposed, which indicate that up to 400 CX gates can be run while still retaining useful quantum information in the signal [43,44,45]. Measurement-conditioned quantum gates are allowed in some higher qubit (≥27) ‘exploratory’ devices provided by the IBM Q. In these devices, therefore, the five-qubit gates of our circuits can be removed, leading to a transpilation with much fewer CX gates. Our future work will investigate the use of these techniques within the quantum routing process.

### 3.3. Discussion

In the experiments of this work, we focused on low-complexity devices since they are likely to be the first to be deployed in real-world quantum networks. However, with a quantum device possessing more qubits, we could potentially bring more benefits for quantum routing schemes. We discuss two of these potential benefits: (i) The quantum router can be extended and generalized to one with more paths. (ii) Different quantum error correction techniques that require a larger number of ancillary qubits can be considered.

Considering (i) above: For a quantum router with *N* paths, *n* control qubits are required, where $N\leq 2^{n}$; and instead of controlled-swap gates, multi-controlled-swap gates are needed. For example, for a quantum router with four paths, ten qubits are required, including two control qubits, one signal qubit, three blank qubits, and four ancillary qubits for the QECC. The principle of a four-path quantum router is similar to the one shown in Figure 1a. The quantum signal is injected into the quantum router via path 1, while the output of the quantum router is entangled with the two control qubits and the four paths. To generate the required entanglement, the quantum router applies three different two-controlled-swap gates: an anti-controlled-controlled-swap gate, a controlled-anti-controlled-swap gate, and a controlled-controlled-swap gate.

## 4. Conclusions

In this work, we experimentally demonstrated an emerging application of quantum entanglement in the communication space. Specifically, we implemented a quantum router on a low-complexity superconducting quantum device. We first bench-marked the quantum router’s performance with all errors being intrinsic to the quantum device only. In comparison with quantum routing on historical NISQ devices, we found that it is critical to have a coupling map that has at least three qubits inter-connected. We also investigated an application of quantum routing—a QRAM implementation—showing its feasibility for both classical and quantum memory states. We then considered noisy quantum signals for evaluating the performance of a five-qubit QECC embedded within the quantum router showing the challenges faced by such error correction. Our experimental results are also consistent with the notion that optimizing the number of CX gates and utilizing measurement error mitigation leads to improved performance in NISQ devices. Our research enlightens the usage of QECCs for today’s quantum devices and points the way forward to the near-term error-corrected quantum routing within real-world quantum networks.

## Figures and Tables

**Table 1:** Syndrome Table with Error Correction Operations.

Syndromes (Error Type ^1^)	Quantum State before Error Correction ^2^	Error Correction ^3^
$0001(X_{2})$, $0101(Y_{2})$, $1010(Z_{3})$, $1100(Z_{5})$	$\alpha_{s}{|0\rangle }_{s}-\beta_{s}{|1\rangle }_{s}$	Z${}_{3}$
$1000(Z_{1})$, $0100(Z_{2})$, $0010(Z_{4})$, $0011(X_{5})$	$-\alpha_{s}{|0\rangle }_{s}-\beta_{s}{|1\rangle }_{s}$	Z${}_{3}$X${}_{3}$Z${}_{3}$X${}_{3}$
$0110(X_{1})$, $1110(Y_{1})$, $0111(X_{3})$, $1011(X_{4})$, $1001(Y_{4})$	$-\alpha_{s}{|1\rangle }_{s}-\beta_{s}{|0\rangle }_{s}$	Z${}_{3}$X${}_{3}$Z${}_{3}$
$1101(Y_{3})$	$-\alpha_{s}{|1\rangle }_{s}+\beta_{s}{|0\rangle }_{s}$	Z${}_{3}$X${}_{3}$
$1111(Y_{5})$	$-\alpha_{s}{|0\rangle }_{s}+\beta_{s}{|1\rangle }_{s}$	X${}_{3}$Z${}_{3}$X${}_{3}$

^1^*X_i_*, *Y_i_*, and *Z_i_* denote the *X*, *Y*, and *Z* errors, respectively, that occurred on the *i*th qubit in the QECC circuit. Note that no error occurred when the syndromes are 0000. ^2^ The quantum state of the third qubit in the QECC circuit after the error-finding. ^3^ X_3_ and Z_3_ represent the X and Z gates applied on the third qubit, respectively

## Data Availability

The data presented in this study are available on request from the corresponding author.

## Appendix A. Tomography Procedure

In this Appendix, we introduce the working principle of quantum state tomography. As a 1-qubit system, $\sigma_{1}$ in the main text can be expressed as [46]

(A1) $$\sigma_{1}=\frac{1}{2}\sum_{i=0}^{3}S_{i}{\hat{\sigma }}_{i}=\frac{1}{2}\left({\hat{\sigma }}_{0}+S_{1}{\hat{\sigma }}_{1}+S_{2}{\hat{\sigma }}_{2}+S_{3}{\hat{\sigma }}_{3}\right).$$

Here, ${\hat{\sigma }}_{0}=I$, ${\hat{\sigma }}_{1}=X$, ${\hat{\sigma }}_{2}=Y$, and ${\hat{\sigma }}_{3}=Z$. The parameters $S_{i}$ are determined by the results of three projective measurements (the X-, Y-, and Z-basis measurements), and the relations are given as

(A2) $$\begin{array}{cc} S_{0} & =P_{Z0}+P_{Z1}=1,\;S_{1}=P_{X0}-P_{X1},\\ S_{2} & =P_{Y0}-P_{Y1},\;\mathrm{and}\;\;S_{3}=P_{Z0}-P_{Z1}, \end{array}$$

where $P_{Z0}$ and $P_{Z1}$ are the probabilities of obtaining the +1 and −1 eigenvalues of the Z operator (the Z-basis measurement), respectively. $P_{X0}$, $P_{X1}$, $P_{Y0}$, and $P_{Y1}$ represent similar probabilities in the X- and Y-basis measurements. Once $S_{i}$ are determined, $\sigma_{1}$ is reconstructed.

$\rho_{1}$ in the main text is a 3-qubit system, which can be given as [46]

(A3) $$\rho_{1}=\frac{1}{8}\sum_{i,j,k=0}^{3}S_{i,j,k}\left({\hat{\sigma }}_{i}⊗{\hat{\sigma }}_{j}⊗{\hat{\sigma }}_{k}\right),$$

where $S_{0,0,0}=1$ due to normalization. Similar to 1-qubit state tomography, 3-qubit state tomography utilizes the results of projective measurements to determine the values of $S_{i,j,k}$. As a result of some redundancy, only 27 projective measurements are required to determine the 63 unknown $S_{i,j,k}$. This redundancy can be verified in Equation (A2), where $S_{0}$ and $S_{3}$ are both determined by the Z-basis measurement results. The 27 projective measurements are tensor products of the Pauli operators on the three qubits, which are {X⊗X⊗X, X⊗X⊗Y, ⋯, Z⊗Z⊗Z}. With known $S_{i,j,k}$, $\rho_{1}$ is reconstructed. The working principle of the 2-qubit state tomography that we utilized in *Experiment-3* is similar.

## Appendix B. Transpilation

Transpilation is a necessary process that rewrites a quantum circuit into a functionally equivalent circuit that can be physically executed on a specific target quantum device and preserves the same measurement outcomes as the original circuit [47]. The transpilation mainly includes three steps: (i) mapping the qubits in the quantum circuit to the chosen quantum device’s topology, (ii) optimizing quantum gates and inserting swap gates to match the topology, and (iii) transforming quantum gates to basis gates that can be physically and directly implemented. The *ibmq_jakarta* only supports five basis gates, as shown in Table A1.

**Table A1:** Basis Gates of the Quantum Device.

Type	Matrix Representation
Single-qubit Gates ^1^	*I* = $\left[\begin{array}{cc} 1 & 0\\ 0 & 1 \end{array}\right]$ , Rz(λ) = $\left[\begin{array}{cc} e^{-i\frac{\lambda }{2}} & 0\\ 0 & e^{i\frac{\lambda }{2}} \end{array}\right]$ , $\sqrt{X}$ = $\frac{1}{2}$ $\left[\begin{array}{cc} 1+i & 1-i\\ 1-i & 1+i \end{array}\right]$ , *X* = $\left[\begin{array}{cc} 0 & 1\\ 1 & 0 \end{array}\right]$
Two-qubit Gate ^2^	CX = $\left[\begin{array}{cccc} 1 & 0 & 0 & 0\\ 0 & 0 & 0 & 1\\ 0 & 0 & 1 & 0\\ 0 & 1 & 0 & 0 \end{array}\right]$

^1^*Rz* is a single-qubit rotation about the Z axis, and *λ* is a phase term. ^2^ Qiskit uses little-endian order.

As the CX gate can only be implemented directly on two physically connected qubits, the transpilation inserts multiple swap gates to move the quantum states of the unconnected qubits (to which a CX gate will be applied) until they are physically adjacent. As each swap gate will be transpiled to three CX gates (which significantly increases quantum gate errors), minimizing the number of swap gates is a critical step for transpilation. However, finding an optimal swap mapping is complicated and prohibitively expensive. By default, Qiskit uses a stochastic algorithm to compute a reasonable swap mapping, a mapping which can be fixed within Qiskit [47].
